# Bioaugmentation with Endophytic Bacterium E6S Homologous to *Achromobacter piechaudii* Enhances Metal Rhizoaccumulation in Host *Sedum plumbizincicola*

**DOI:** 10.3389/fpls.2016.00075

**Published:** 2016-02-04

**Authors:** Ying Ma, Chang Zhang, Rui S. Oliveira, Helena Freitas, Yongming Luo

**Affiliations:** ^1^Key Laboratory of Soil Environment and Pollution Remediation, Institute of Soil Science, Chinese Academy of SciencesNanjing, China; ^2^Centre for Functional Ecology, Department of Life Sciences, University of CoimbraCoimbra, Portugal; ^3^Chuzhou UniversityChuzhou, China; ^4^Centro de Biotecnologia e Química Fina – Laboratório Associado, Escola Superior de Biotecnologia, Universidade Católica PortuguesaPorto, Portugal; ^5^Department of Environmental Health, Research Centre on Health and Environment, School of Allied Health Sciences, Polytechnic Institute of PortoVila Nova de Gaia, Portugal; ^6^Yantai Institute of Coastal Zone Research, Chinese Academy of SciencesYantai, China

**Keywords:** endophytic bacterium, rhizoaccumulation, multi-metal contamination, phytostabilization, *Sedum plumbizincicola*

## Abstract

Application of hyperaccumulator-endophyte symbiotic systems is a potential approach to improve phytoremediation efficiency, since some beneficial endophytic bacteria are able to detoxify heavy metals, alter metal solubility in soil, and facilitate plant growth. The objective of this study was to isolate multi-metal resistant and plant beneficial endophytic bacteria and to evaluate their role in enhancing plant growth and metal accumulation/translocation. The metal resistant endophytic bacterial strain E6S was isolated from stems of the Zn/Cd hyperaccumulator plant *Sedum plumbizincicola* growing in metalliferous mine soils using Dworkin and Foster salts minimal agar medium with 1-aminocyclopropane-1-carboxylate (ACC) as the sole nitrogen source, and identified as homologous to *Achromobacter piechaudii* based on morphological and biochemical characteristics, partial 16S rDNA sequence and phylogenetic analysis. Strain E6S showed high level of resistance to various metals (Cd, Zn, and Pb). Besides utilizing ACC, strain E6S exhibited plant beneficial traits, such as solubilization of phosphate and production of indole-3-acetic acid. Inoculation with E6S significantly increased the bioavailability of Cd, Zn, and Pb in soil. In addition, bacterial cells bound considerable amounts of metal ions in the following order: Zn > Cd >Pb. Inoculation of E6S significantly stimulated plant biomass, uptake and bioaccumulation of Cd, Zn, and Pb. However, E6S greatly reduced the root to shoot translocation of Cd and Zn, indicating that bacterial inoculation assisted the host plant to uptake and store heavy metals in its root system. Inoculation with the endophytic bacterium E6S homologous to *A. piechaudii* can improve phytostabilization of metalliferous soils due to its effective ability to enhance *in situ* metal rhizoaccumulation in plants.

## Introduction

Mining activities produce waste tailings containing high levels of metal pollutants that have significant environmental impacts and can affect human health through the food chain (Moreno et al., 2010). During mineral ore processing mine tailings are generated and mostly left without proper management, therefore leading to metal contamination of surrounding soils, with detrimental impacts on the soil microbial community and consequent reduction in ecosystem functioning (Kossoff et al., 2014).

Phytoremediation, application of plants to remove (phytoextraction), stabilize (phytostabilization), or volatilize (phytovolatilization) heavy metals *in situ* in a more attractive and cost-effective manner than the conventional physicochemical technologies, has received increasing attention over the last decades (Raskin and Ensley, 2000). Particularly, phytostabilization of metal contaminated mine tailings, which uses plants that minimize metal accumulation into aboveground tissues, seems to be most promising for remediating polluted sites when phytoextraction is not a feasible option (Mendez and Maier, 2008). Because mine tailings have low nutrient contents, to overcome the limitations of plant establishment, soil amendments with fresh or composted organic matter (biochemical amendments) can enhance plant colonization and reduce metal toxicity and solubility, thereafter improving phytostabilization efficiency (Lee et al., 2011). However, most of those biochemical amendments, such as cyclonic ashes, steel shots. and superphosphate, are toxic to plants and their associated microbes (Ribeiro Filho et al., 2011). Some beneficial bacteria have been successfully employed for environmental applications (so-called bioaugmentation) due to their ability to: (1) promote plant growth by producing beneficial metabolites [e.g., siderophores, indole-3-acetic acid (IAA) and 1-aminocyclopropane-1-carboxylate (ACC) deaminase] and solubilizing phosphate (P); and/or (2) alter soil metal mobility without disturbing soil ecological structure and function through various mechanisms such as metal biosorption/bioaccumulation, redox reaction, chelation, or complexation (Glick, 2010; Ma et al., 2011a). Amongst all the beneficial features, the production of ACC deaminase is considered as one of the major plant growth promoting traits of bacteria (Ma et al., 2011a). Mesa et al. (2015) reported that the inoculation of autochthonous rhizobacteria stimulated the biomass of native *Spartina maritima*, and enhanced metal (As, Cu, Pb, and Zn) accumulation in the root of plants (rhizoaccumulation) grown in multi-metal contaminated soils. Since bacterial endophytes have more intimate association with host plants than rhizobacteria, they could be reliable bioinoculants for improving metal phytostabilization.

Although the effects of inoculating endophytic bacterial strains on growth promotion of various host and/or non-host plants have been reported (Ma et al., 2011b; Shin et al., 2012; Visioli et al., 2014), little is known on application of endophytes for enhanced phytoremediation of natural non-sterile polluted soils. *Sedum plumbizincicola* is known to hyper-accumulate or extract Cd and Zn from soils (Jiang et al., 2010). Despite the potential of *S. plumbizincicola* to remove various metals from polluted soil, its slow growth is a limitation that needs to be overcome.

The objectives of this study were to: (1) isolate and characterize metal resistant endophytic bacteria that can utilize ACC as the sole nitrogen (N) source; (2) assess plant beneficial activities, metal biosorption and mobilization capacities of a selected endophytic bacterium; (3) examine the effect of the endophytic bacterium on plant growth and metal accumulation/translocation in host *S. plumbizincicola* in non-sterile multi-metal contaminated soils.

## Materials and Methods

### Isolation and Identification of ACC-Utilizing Endophytic Bacterium

Bacterial strains were isolated from tissues of Zn/Cd hyperaccumulator *S. plumbizincicola* growing on metalliferous mine soils in Chunan city of Zhejiang, Southeast of China. Plant samples were washed thoroughly with tap water followed by three rinses with deionized water and then separated into roots, stems, and leaves. Plant organs were sterilized by immersion for 1 min in 70% (v/v) ethanol, and then 3% sodium hypochlorite for 3 min and washed three times with sterile water to remove residual chemicals. To confirm the success of the surface sterilization process, plant tissues were plated on Luria–Bertani (LB) agar plate to detect epiphytic bacteria. No contamination was found. The plant tissues (0.5 g) were ground with a mortar and pestle in 5 mL of sterile deionized water. The appropriate dilutions were plated onto sucrose-minimal salts low-phosphate agar medium supplemented with 100 mg L^-1^ of Cd (CdCl_2_), Zn (ZnSO_4_), and Pb [Pb(NO_3_)_2_]. After incubating at 26°C for 5 days, colonies were randomly picked based on distinct colony morphology, purified and re-streaked on the same media. To isolate beneficial endophytes, the growth of all metal resistant isolates was evaluated on Dworkin and Foster salts minimal medium (Dworkin and Foster, 1958) containing ACC as the sole N source.

One bacterial isolate which showed fast growth and capability of utilizing ACC as the sole N source was identified and used for further study. Total DNA was extracted from cells of the selected bacterial isolate using the QuickExtract^TM^ bacterial DNA extraction kit. The 16S ribosomal gene was amplified using the primers 27F (5′-AGAGTTTGATCCTGGCTCAG-3′) and 1492R (5′-GGTTACCTTGTTACGACTT-3′) under the reaction conditions described by Branco et al. (2005). The amplification product (5 μL) was separated by agarose gel (1%, w/v) electrophoresis in TAE buffer (0.04 M Tris acetate, 1 mM EDTA) containing 1 μg mL^-1^ ethidium bromide. Partial nucleotide sequence of the amplified 16S rDNA was determined with automated DNA sequencer and then compared with similar sequences in the GenBank using BLAST.

### Biochemical Characterization of Endophytic Bacterium

The bacterial isolate was grown for 5 days at 26°C on LB media supplemented with heavy metal (Cd, Zn, and Pb) at varying concentrations (50–1500 mg L^-1^). The highest concentration of metal allowing bacterial growth was defined as its resistance level. The antibiotic sensitivity of the isolate was determined by the disk diffusion method (Rajkumar et al., 2008).

The ACC deaminase activity of the isolate was examined by monitoring the concentration of α-ketobutyrate (α-KB) generated through the enzymatic hydrolysis of ACC as described by Honma and Shimomura (1978). The protein content of cell suspensions was determined by the Bradford method (Bradford, 1976). Synthesis of IAA by the strain was assayed as described by Bric et al. (1991) using LB medium with 0.5 mg mL^-1^ of *L*-tryptophan. Bacterial siderophore production was detected by chrome azurol S (CAS) agar plate assay (Schwyn and Neilands, 1987). The P solubilization by the isolate was analyzed in modified Pikovskayas medium (Sundara-Rao and Sinha, 1963) and the solubilized P in the culture supernatant was determined as described by Park et al. (2011).

### Metal Mobilization and Biosorption Analyses

In the metal mobilization assay, the physicochemical properties of contaminated agricultural soil collected from Fuyang city of Zhejiang Province, China were: pH (1:1 w/v water) 8.1; organic matter 36.3 g kg^-1^; cation exchange capacity 11.4 cmol kg^-1^; Cd 5.9 mg kg^-1^; Zn 736 mg kg^-1^; Pb 153 mg kg^-1^. The soil was air dried, crushed (<2 mm) and then autoclaved at 121°C for 2 h. The bacterial strain was incubated at 27°C with shaking at 200 rpm for 18 h. After adjusting cell density to an OD_600_ of 1.5, 5 mL of culture were centrifuged, then gently washed twice with 0.05 M phosphate buffer (pH 7.2) and three times with sterile deionized water, recentrifuged and finally resuspended in 5 mL sterile deionized water. One milliliter of washed bacterial culture at an OD_600_ of 1 (treatment) or sterile deionized water (control), was added to 1 g of sterilized soil in 50 mL sealed centrifuge tubes. All tubes were weighed and kept at 27°C with 200 rpm in the dark. The total weight was kept constant by adding sterile deionized water to compensate for evaporation. After 7 days, 10 mL of sterile deionized water were added to extract water-soluble metal from soil. The suspensions were centrifuged at 7000 rpm for 10 min and filtered. Metal (Cd, Zn, and Pb) contents of bioavailable fraction in each filtrate were determined using a flame atomic absorption spectrophotometer (AAS; Ma et al., 2011b).

The biosorption of Cd, Zn, and Pb by bacterial cells was evaluated as described by Rajkumar et al. (2009). The bacterium was grown to exponential (OD_600_ = 1) at 27°C in LB medium. Cells were harvested by centrifugation at 6000 rpm for 20 min and washed twice with sterile deionized water. The wet biomass was re-suspended in 150 mg L^-1^ of Cd, Zn, or Pb. Cells were harvested after incubation at room temperature for 10 h by centrifugation and the residual metal ion in the supernatant was measured by AAS. The amount of metal biosorbed onto bacterial cells was calculated by subtracting the metal concentration in the supernatant from the original concentration.

### Microcosm Experiments

The selected ACC-utilizing strain was initially assessed for its ability to promote plant growth in phytagar media supplied with or without 10 mg Cd L^-1^ using *Brassica napus* as a model plant (Ma et al., 2011b). For pot experiment, the multi-metal contaminated soil described above was used, while agricultural soil from Nanjing, China was used for comparison. The soil was air dried, sieved (2 mm), and stored at 20°C before use. *S. plumbizincicola* seedlings of equal size were obtained from an old Pb/Zn mine in the Zhejiang province of China. Shoot samples with ca. 5 cm were well cleaned with tap water and grown hydroponically in half-strength Hoagland’s nutrient solution for a week. Before inoculation, bacterial colony marked with antibiotic resistance was obtained after incubating parental strain onto LB agar containing ampicillin (100 mg L^-1^) and tetracycline (75 mg L^-1^), simultaneously. Surface sterilized roots of precultured seedlings were soaked for 2 h in the bacterial culture (OD_600_ of 1.5) or sterile water (controls) and transplanted into 1 L pot filled with 750 g of soil. The seedlings (six plants pot^-1^) were allowed to grow under greenhouse condition (25 ± 5°C, 16:8 day/night regime). Each treatment was performed in five replicates. After 75 days, plants were carefully removed from the pots and the root surface was cleaned thoroughly with deionized water. Plant root and shoot length, fresh and dry weight were measured. The concentrations of Cd, Zn, and Pb in root and shoot were quantified as described by Ma et al. (2009). The translocation factor (TF) was calculated as the ratio of metal content in the shoots to that in the roots (Malik et al., 2010) and the bioaccumulation factor (BCF) was calculated as the ratio of metal contents in the entire plant to that in the soil (Abdul and Thomas, 2009). The colonization of introduced strain in the rhizosphere and interior tissues of *S. plumbizincicola* was determined using the intrinsic antibiotic marker combined with the dilution-plate method (Ma et al., 2011b).

### Statistical Analysis

Student’s *t*-test (*p* < 0.05) or Analysis of Variance (ANOVA) followed by the *post hoc* Fisher Least Significant Difference test (*p* < 0.05) were used to compare treatment means. All the statistical analyses were performed using SPSS 17.0.

## Results and Discussion

### Isolation and Identification of Metal Resistant and ACC-Utilizing Endophytic Bacterium

Although the interaction between endophytic bacteria and their host plants is not completely understood, some endophytic bacteria isolated from heavy metal hyperaccumulators appear to exert beneficial effects on their hosts (Shin et al., 2012), such as amelioration of metal stress, stimulation of plant establishment and growth, and biocontrol of phytopathogens (Ma et al., 2011a). Consequently, such beneficial endophytic bacteria could be isolated and selected for their application in assisting phytoremediation of metal contaminated soils (Rajkumar et al., 2009). In this study, we isolated a metal resistant endophytic bacterial strain from stems of Zn/Cd hyperaccumulator *S. plumbizincicola* and assessed its effect as a bioinoculant on multi-metal phytoremediation by its host plant. The initial screening was based on the morphological differences of bacterial colonies and resulted in the isolation of 42 metal (100 mg L^-1^ of Cd, Zn, and Pb) resistant endophytic strains from interior tissues of *S. plumbizincicola*. Out of 42 strains, isolate E6S was specifically chosen based on its fast growth and capability of utilizing ACC as the sole N source.

**FIGURE 1:**
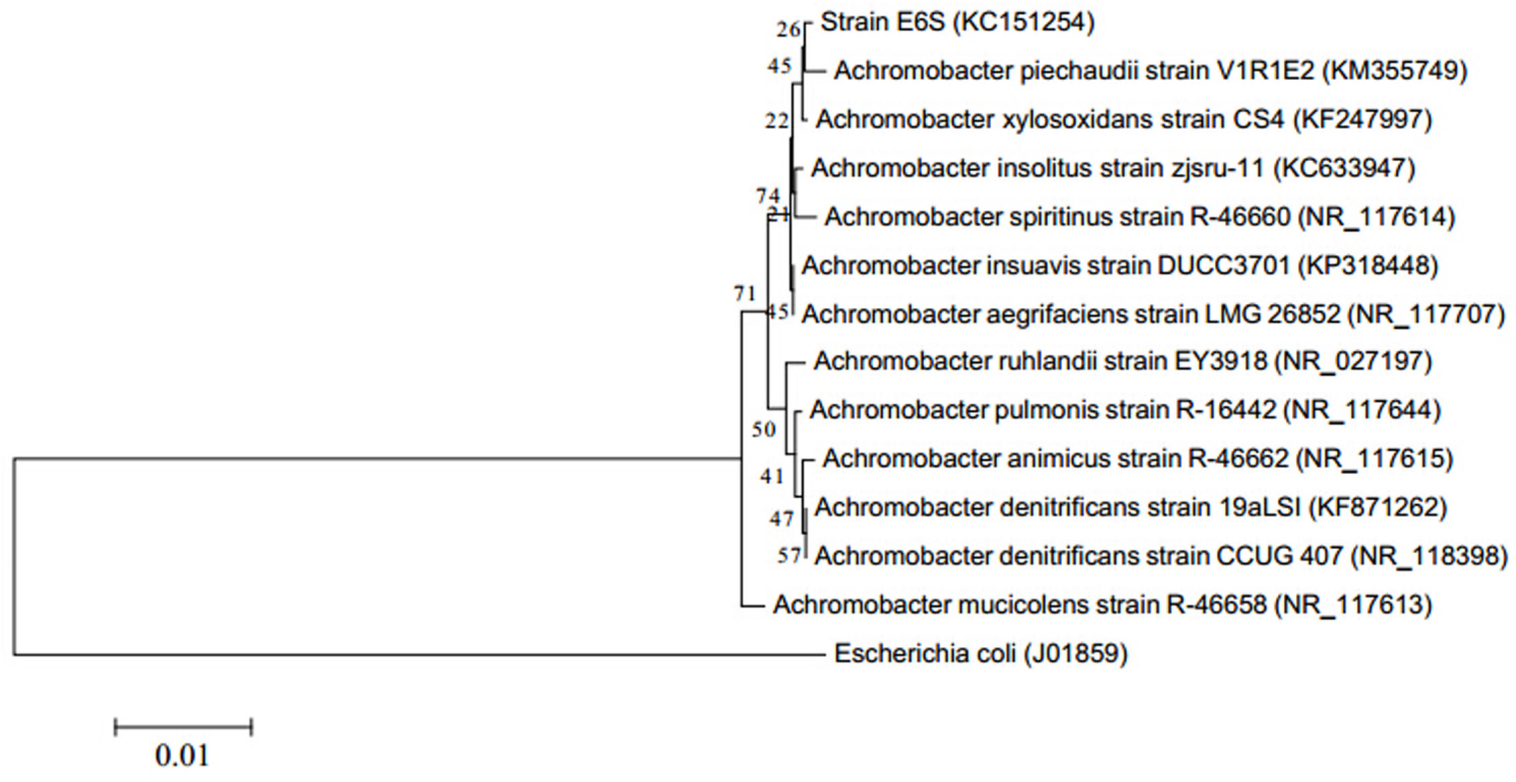
**Phylogenetic tree showing the relationship of partial 16S rDNA gene sequences from endophytic bacterium E6S homologous to *Achromobacter piechaudii* with other related sequences from *Achromobacter*.** *Escherichia coli* was used as the out-group. The value on each branch is the percentage of bootstrap replications supporting the branch.

Based on morphological and biochemical characteristics (**Table 1**), comparative analysis of 16S rDNA sequence and phylogenic analysis (**Figure 1**), strain E6S was identified as being homologous to *Achromobacter piechaudii* (100% similarity). The sequence obtained (858 bp) was submitted to the NCBI databases under the accession number KC151254. Strain E6S was gram-negative, motile, rod shaped and positive for oxidase and catalase. It was able to produce H_2_S, utilize citrate and hydrolyze starch (**Table 1**).

**Table 1:** Morphological, physiological, and biochemical characteristics of endophytic bacterium E6S homologous to *Achromobacter piechaudii*.

Characteristic	E6S
Gram staining	–
Cell shape	Rod
Oxygen requirements	Aerobic
Motile	+
Growth at 15–40°C	+
Growth at 6% NaCl	+
Oxidase	+
Catalase	+
Indole production	–
Voges–Proskauer test	–
H_2_S production	+
Nitrate reduction	+
Nitrite reduction	–

Utilization of Arabinose	–
Mannitol	–
Maltose	–
Glucose	–
Citrate	+
Lactate	–

Hydrolysis of Casein	–
Starch	+
Gelatin	–
Esculin	–


*+, positive; –, negative.*

### Biochemical Properties of Endophytic Bacterium

Under various abiotic stresses (e.g., heavy metals, drought, and salinity), microorganisms face a constant battle for limited resources and try to adapt to unfavorable environmental conditions by acting as stress ameliorators (Hibbing et al., 2010). Hence, strain E6S was tested for the ability to grow on both metal and antibiotic-supplemented agar media. Strain E6S was found to exhibit resistance to heavy metal (Cd, Zn, and Pb) and various antibiotics (**Table 2**). The order of the toxicity of metals to the isolate was found to be Cd > Zn > Pb. This strain exhibited high tolerance to multiple metals, which could be attributed to the fact that it was isolated from tissues of a Zn/Cd hyperaccumulator plant, likely containing high levels of these bioavailable metal ions (Idris et al., 2004). Among six antibiotics tested, strain E6S showed resistance to ampicillin and tetracycline.

**Table 2:** Heavy metal tolerance, antibiotic resistance, and plant growth promoting traits of endophytic bacterium E6S homologous to *Achromobacter piechaudii*.

Parameter	Unit	E6S
Metal tolerance	mg L^-1^	
Cd		300
Zn		750
Pb		1200

Antibiotic resistance	mm	
Ampicillin (10 μg)		3 (R)
Tetracycline (30 μg)		9 (R)
Streptomycin (20 μg)		25 (S)
Chloramphenicol (30 μg)		15 (I)
Kanamycin (30 μg)		23 (S)

Plant growth promoting traits		
ACC deaminase	μm α-KB mg^-1^ h^-1^	11 ± 0.9
P solubilization	mg L^-1^	135 ± 16
IAA production	mg L^-1^	22 ± 6
Siderophore (CAS)	cm	nd


*R, resistant (<10 mm); I, intermediate (10–15 mm); S, susceptible (>15 mm); ACC deaminase, 1-aminocyclopropane-1-carboxylate deaminase; α-KB, α-ketobutyrate; P, phosphate; IAA, indole-3-acetic acid; CAS, chrome azurol S; nd, not detected.*

Endophytic bacteria can stimulate plant growth directly by solubilizing unavailable nutrients (e.g., P, N, and potassium), sequestering iron by siderophores and phytohormones (e.g., IAA) or indirectly by inducing a systemic resistance in plants against various types of pathogens (Ma et al., 2011b). These features make them perfect choices for improving phytoremediation. Strain E6S was able to produce ACC deaminase, IAA and solubilize P, whereas no biosynthesis of siderophore was found (**Table 2**). In general, bacterial IAA at low level has been implicated in promoting primary root elongation through cell division, however, a high IAA level can inhibit primary root growth but stimulate lateral root formation (Gravel et al., 2007). A low level of IAA production by strain E6S (22 mg L^-1^) suggests close relationship between plant growth promotion activity (**Table 3**) and IAA production. Strain E6S exhibited high tricalcium phosphate-solubilizing ability (135 mg L^-1^; **Table 2**), which may compensate for P deficiency-induced plant growth retardation in metal contaminated soil. ACC-utilizing bacteria have been found to prevent the inhibition of root growth by hydrolyzing the ethylene precursor ACC into ammonia and α-KB and inhibiting ACC synthase activity (Arshad et al., 2007). Although strain E6S showed relatively low ACC deaminase activity (11 μm α-KB mg^-1^ h^-1^), it seemed to be efficient in promoting plant growth under metal stress (**Table 3**). The results suggest that the endophytic bacteria, which can synthesize beneficial metabolites such as IAA, ACC deaminase, and solubilize P should be considered as promising biofertilizers.

**Table 3:** Effects of endophytic bacterium E6S homologous to *Achromobacter piechaudii* on the growth of *Brassica napus* in phytagar assay and *Sedum plumbizincicola* in pot experiment.

Type	Treatment		Relative elongation ratio (RER) of root^a^	RER of shoot^b^	Fresh weight (mg)	Dry weight (mg)	Ratio of root/shoot dry weight^c^
Phytagar assay	Control	Blank	–	–	53.5 ± 3.1 b	2.6 ± 0.0 b	0.74 ± 0.08 b
		E6S	1.62 ± 0.18 b	1.15 ± 0.10 b	67.6 ± 5.3 a	3.2 ± 0.3 a	1.04 ± 0.08 a
	10 mg L^-1^ Cd	Blank	–	–	28.8 ± 4.4 c	1.9 ± 0.3 c	0.32 ± 0.04 c
		E6S	2.78 ± 1.00 a	1.54 ± 0.20 a	47.6 ± 4.2 b	2.8 ± 0.2 b	0.62 ± 0.03 b

Pot experiment	Pristine soil	Blank	–	–	1735 ± 169 z	221 ± 16 z	0.09 ± 0.05 w
		E6S	1.26 ± 0.13 x	1.27 ± 0.10 w	2416 ± 157 y	275 ± 19 y	0.12 ± 0.01 w
	Metal polluted soil	Blank	–	–	6086 ± 403 x	739 ± 24 x	0.02 ± 0.01 x
		E6S	1.89 ± 0.16 w	1.36 ± 0.10 w	8211 ± 481 w	946 ± 51 w	0.03 ± 0.01 x


*Average ± standard deviation from five samples. Data of columns indexed by the same letter within each treatment (with or without Cd; pristine or polluted soil) are not significantly different between bacterial treatments according to Fisher’s least significant difference (LSD) test (*p* < 0.05).*

*^a^RER of root = Mean root length of tested plant/Mean root length of control × 100.*

*^b^RER of shoot = Mean shoot length of tested plant/Mean shoot length of control × 100.*

*^c^Ratio of root/shoot dry weight = Dry weight of root/Dry weight of shoot.*

### Metal Mobilization and Biosorption Potential of Endophytic Bacterium

**FIGURE 2:**
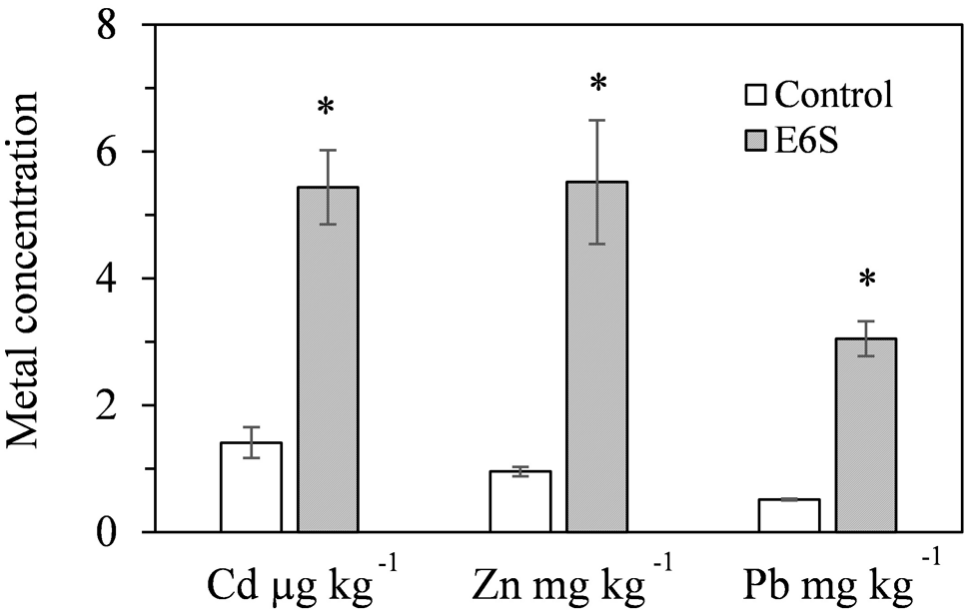
**Effect of inoculation with E6S on the mobilization of Cd, Zn, and Pb in soil.** Bars represent standard deviations of triplicates. An asterisk (^∗^) denotes a value significantly greater than the corresponding control value according to Student’s *t*-test (*p* < 0.05).

Endophytic bacterium E6S homologous to *A. piechaudii* displayed the potential for biological mobilization of Cd, Zn, and Pb in multi-metal contaminated soil (**Figure 2**). The inoculation of E6S significantly increased (*p* < 0.05) water extractable Cd, Zn, and Pb in metal contaminated soils by 3.9-, 5.8- and 6.0-fold, respectively, compared to the controls. The observation indicates that strain E6S facilitated the release of metals from non-soluble phase in the soil matrix, thus improving their bioavailability to plants. This may be attributed to organic acid production and phosphate solubilization mediated reduction in soil pH (Ma et al., 2009, 2015).

**FIGURE 3:**
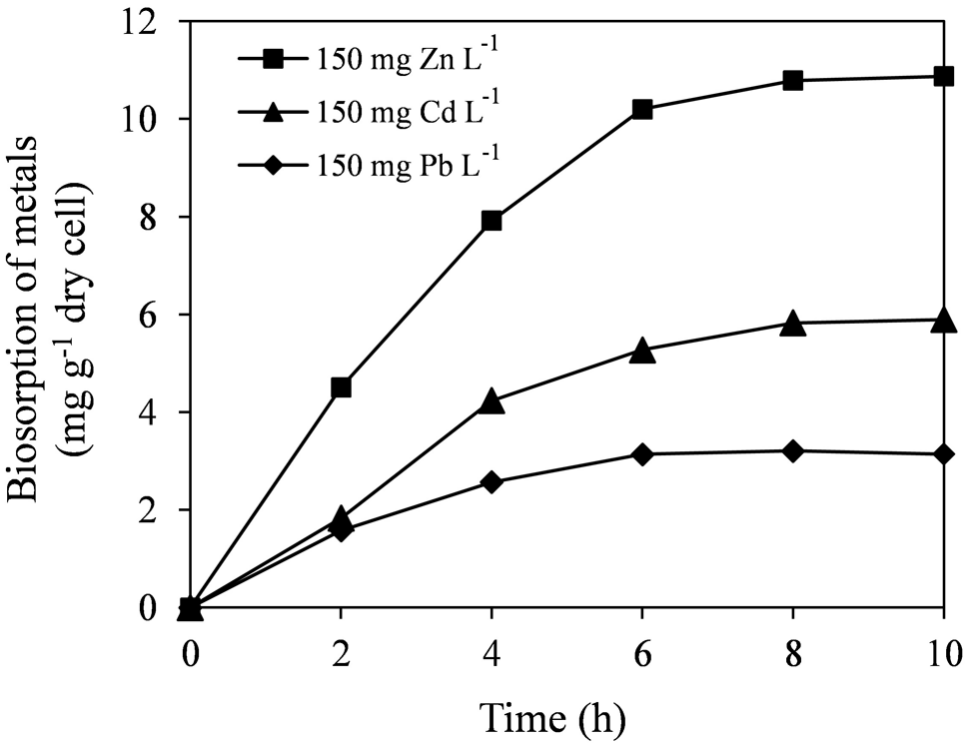
**Biosorption of Cd, Zn, and Pb on E6S cells.** Bars represent standard deviations of triplicates.

Biosorption capacity of bacteria plays an important role in reducing metal phytotoxicity by limiting the entry of metal ions into plant cells and may contribute for enhanced plant growth in metal contaminated soils (Ma et al., 2011a). At 150 mg L^-1^ initial metal concentrations, strain E6S was able to remove significant amounts of Cd, Zn, or Pb within 10 h incubation (**Figure 3**). After 8 h incubation, maximum biosorption by E6S was reached, thereafter remaining constant. This was probably due to the achievement of specific equilibrium for metal concentrations. The highest content of metal biosorption was observed with Zn (10.9 mg g^-1^ of cell dry weight), while the lowest was seen with Pb (3.2 mg g^-1^ of cell dry weight). This was probably due to ionic radius of each metal ion (Karakagh et al., 2012), since Zn (0.88 Å) with smaller ionic radius may be more rapidly complexed by bacterial cell wall/membrane compared with Cd (0.97 Å) and Pb (1.2 Å).

### Effects of Endophytic Bacterial Inoculation on Plant Biomass

In phytagar assay, inoculation of E6S induced significant increases in fresh and dry weight, ratio of root/shoot dry weight of *B. napus* in both unpolluted and polluted (10 mg L^-1^ Cd) phytagar media (**Table 3**). For instance, E6S induced an increase in fresh and dry weight, and ratio of root/shoot dry weight in Cd polluted media by 65, 47, and 94%, respectively, compared to non-inoculated control. Bacterial inoculation greatly enhanced the biomass production of *B. napus* under non-stressed and metal-stressed conditions. Plants inoculated with strain E6S presented a significantly higher root/shoot ratio than the respective controls, indicating that with the help of endophytic bacterium E6S, plants can acquire nutrients more efficiently from soils for plant biomass production, especially for roots. Moreover, the relative elongation rate (RER) of root and shoot in unpolluted media (1.62 and 1.15) were lower than that in polluted treatment (2.78 and 1.54). A possible explanation might be that the endophytic bacteria could exert more efficient functions that help plants to cope with adverse environmental stress (Rajkumar et al., 2009).

In pot experiment, inoculation of E6S significantly improved fresh and dry weight of *S. plumbizincicola* in both pristine and multi-metal polluted soils under non-sterile conditions (**Table 3**). For example, inoculation of E6S considerably increased plant fresh and dry weight by 35 and 28%, respectively, in metal polluted soils. The beneficial effects of E6S on growth of host *S. plumbizincicola* were also observed on growth of non-host *B. napus* in phytagar assay. These effects may be attributed to beneficial metabolites produced by strain E6S, such as IAA and ACC deaminase, which alleviated metal phytotoxicity and thereafter stimulated plant development. Additionally, inoculation of E6S did not significantly influence ratio of root/shoot dry weight in neither pristine nor polluted soils. The presence of metals declined ratio of root/shoot dry weight, compared with non-polluted control. These results concur with the earlier observations of Wan et al. (2012) that elevated metal concentrations mainly impaired root growth, in spite of *S. plumbizincicola* being qualified as a Zn/Cd hyperaccumulator. It is therefore concluded that bioinoculant E6S could serve as biofertilizer for phytoremediation purposes. Interestingly, the fresh and dry weights of *S. plumbizincicola* grown in metal contaminated soil were higher than in pristine soil (**Table 3**). A possible explanation could be that the current levels of heavy metals in soil are not toxic to the plant and can stimulate its growth. This may be attributed to both a beneficial effect of improved metal nutrition and/or the activation of stress scavenging mechanisms, which can help the plant to cope with environmental stresses (Dalcorso et al., 2013).

### Endophytic Bacteria-Enhanced Phytostabilization

The changes in metal solubility in polluted soils caused by biological amendments can contribute to facilitate metal accumulation in plants (Ma et al., 2009). Therefore, the effects of metal mobilizing bacterium E6S on metal uptake and translocation by *S. plumbizincicola* were evaluated. In general, inoculation of strain E6S significantly improved plant uptake of Cd, Zn, and Pb (**Table 4**). For instance, strain E6S increased Cd, Zn, and Pb concentration in *S. plumbizincicola* by 32, 37, and 89%, respectively, which is in accordance with significant improvements of BCF of metal (Cd, Zn, and Pb) induced by E6S. This corroborates the data shown in **Figure 2** for bacterial metal mobilization, indicating that inoculation of E6S facilitated metal (Cd, Zn, and Pb) bioavailability in soils and thereby their uptake by plants. However, the bacterial inoculation significantly decreased TF of Cd and Zn (*p* < 0.05; **Table 4**) and metal accumulation in shoots (data not shown). The TF was 2.7 and 1.5 (>1) for Cd and Zn in non-inoculated plants, but it decreased to 0.5 and 0.7 (<1) after inoculation with strain E6S. *S. plumbizincicola* has been reported to be a hyperaccumulator for Cd and Zn phytoextraction due to their TF > 1, however, our data showed that the inoculation of strain E6S inhibited the plant-self translocation of metal from roots to shoots and helped plants store metals in their roots, which is desirable for phytostabilization purposes. The present observations indicate that strain E6S effectively increased the bioavailability of metal (Cd, Zn, and Pb) in the rhizosphere soils and also promoted the growth of host *S. plumbizincicola* plants, consequently increasing the total plant metal uptake, while diminishing the translocation of metals from roots to shoots. Results suggest that endophytic bacterium E6S can not only protect the plants against the inhibitory effects of multiple metals, but also effectively improve rhizoaccumulation of toxic metals. Therefore, it can be used to assist phytostabilization of heavy metals in the plant root system. Previously, Srivastava et al. (2013) also reported that *Staphylococcus arlettae* NBRIEAG-6 enhanced As accumulation in roots of *B. juncea* and helped in As phytostabilization. Recently, Sura-de Jong et al. (2015) reported that selenium hyperaccumulators harbor a diverse endophytic bacterial community. It is becoming apparent that some endophytic bacteria play an important role in metal hyperaccumulation and translocation processes, contributing to phytoextraction, while others seem to be effective in improving rhizoaccumulation, contributing to phytostabilization. Future studies are, therefore, needed to access the structure and diversity of bacterial endophytes of *S. plumbizincicola* and their influence in phytoremediation processes.

**Table 4:** Effects of endophytic bacterium E6S homologous to *Achromobacter piechaudii* on metal uptake, translocation factor and bioaccumulation factor by *Sedum plumbizincicola*.

Treatment	Cd			Zn			Pb		
			
	Plant uptake (mg kg^-1^ dw)	TF	BCF	Plant uptake (mg kg^-1^ dw)	TF	BCF	Plant uptake (mg kg^-1^ dw)	TF	BCF
Control	127 ± 8	2.7 ± 0.3^∗^	21.6 ± 1.4	2262 ± 61	1.5 ± 0.1^∗^	3.1 ± 0.1	114 ± 10	0.2 ± 0.0	0.7 ± 0.1
E6S	168 ± 25^∗^	0.5 ± 0.1	28.4 ± 4.2^∗^	3095 ± 380^∗^	0.7 ± 0.1	4.9 ± 0.2^∗^	215 ± 20^∗^	0.2 ± 0.0	1.4 ± 0.1^∗^


*Average ± standard deviation from five samples. ^∗^A value significantly greater than the corresponding control according to Student’s t-test (*p* < 0.05). TF, translocation factor; BCF, bioaccumulation factor; dw, dry weight.*

### Bacterial Colonization

After 75 days of inoculation onto plants, strain E6S exhibited high-density colonization of the rhizosphere [3.9 × 10^5^ colony-forming units (CFU) g^-1^], and fresh roots, stems and leaves (5.4, 3.7, and 0.8 × 10^3^ CFU g^-1^, respectively) of *S. plumbizincicola*, indicating the great potential of this strain to establish, survive, and develop inside plant tissues and in the root zone. Since beneficial bacteria have great potential to contribute to sustainable plant growth promotion and metal rhizoaccumulation, the colonization and survival properties of introduced beneficial strains are crucial features to evaluate the capacity to assist their host plants in coping with metal stress and therefore phytostabilization efficiency in contaminated sites (Ma et al., 2011a).

## Conclusion

Endophytic bacterium E6S homologous to *A. piechaudii* isolated from metal hyperaccumulator *S. plumbizincicola* stems was able to facilitate plant growth through the production of IAA and ACC deaminase and solubilization of P. The characterization studies showed that the isolate resisted high concentrations of Cd, Zn, and Pb, via extracellular biosorption in metal containing liquid media and increased water extractable metal concentration in metal contaminated soils. The inoculation of E6S significantly enhanced plant growth and rhizoaccumulation of Cd, Zn, and Pb by host *S. plumbizincicola*, however, the TFs of Cd and Zn remarkably declined in the presence of bacterial inoculation. Effective metal biosorption and mobilizing capacities as well as the potential to adapt to/survive multi-metal stress conditions along with various plant beneficial traits are clear indications of the advantages of employing this microorganism as a bioinoculant for ameliorating metal phytotoxicity and thus enhancing phytostabilization efficiency. Further work will address the mechanism of the selected bacterial strain contributing to reduce metal translocation from roots to shoots and its effect on the plant biomass yield and metal phytostabilization in field experiments.

## Author Contributions

YM wrote the manuscript and carried out experiments; CZ and RO analyzed experimental results; HF was the project sponsor; YL designed experiments.

## Conflict of Interest Statement

The authors declare that the research was conducted in the absence of any commercial or financial relationships that could be construed as a potential conflict of interest.
